# QSAR‐Based Estimation of Species Sensitivity Distribution Parameters: An Exploratory Investigation

**DOI:** 10.1002/etc.4601

**Published:** 2019-11-09

**Authors:** Renske P.J. Hoondert, Rik Oldenkamp, Dick de Zwart, Dik van de Meent, Leo Posthuma

**Affiliations:** ^1^ RIVM, Centre for Sustainability Environment and Health Bilthoven The Netherlands; ^2^ Department of Environmental Sciences, Faculty of Science Radboud University Nijmegen Nijmegen The Netherlands; ^3^ ARES Odijk The Netherlands

**Keywords:** Ecotoxicity data, No‐observed‐effect concentration, Median effect concentration, Species sensitivity distribution parameters

## Abstract

Ecological risk assessments are hampered by limited availability of ecotoxicity data. The present study aimed to explore the possibility of deriving species sensitivity distribution (SSD) parameters for nontested compounds, based on simple physicochemical characteristics, known SSDs for data‐rich compounds, and a quantitative structure–activity relationship (QSAR)‐type approach. The median toxicity of a data‐poor chemical for species assemblages significantly varies with values of the physicochemical descriptors, especially when based on high‐quality SSD data (from either acute median effect concentrations or chronic no‐observed‐effect concentrations). Beyond exploratory uses, we discuss how the precision of QSAR‐based SSDs can be improved to construct models that accurately predict the SSD parameters of data‐poor chemicals. The current models show that the concept of QSAR‐based SSDs supports screening‐level evaluations of the potential ecotoxicity of compounds for which data are lacking. *Environ Toxicol Chem* 2019;38:2764–2770. © 2019 The Authors. *Environmental Toxicology and Chemistry* published by Wiley Periodicals, Inc. on behalf of SETAC

## Graphical abstract

Extrapolation of data for species sensitivity distribution (SSD) parameters (mu and sigma) using a QSAR approach (estimating SSD parameters based on physicochemical characteristics) may in the end result in filling in data gaps for ‐ in theory ‐ all chemicals. QSAR = quantitative structure–activity relationship; SSD = species sensitivity distribution.

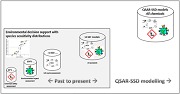

## INTRODUCTION

A recent publication presented species sensitivity distributions (SSDs) for 12 386 compounds, with potential utility for prevention, assessment and management of chemical pollution (Posthuma et al. 2019). Chemical pollution is an environmental stress factor with growth in both numbers of compounds and masses produced and emitted (Bernhardt et al. 2017; United Nations Environmental Programme 2019). Because there are more than 100 000 compounds in commerce (European Chemicals Agency 2019) and there has been further growth, there is a need for a continuous expansion of impact‐assessment methods, to preferably cover all compounds. The present study aimed to explore whether and how far the SSD parameters of nontested compounds can be estimated from physical and chemical molecular descriptors, by means of a quantitative structure–activity relationship (QSAR) approach.

The working hypothesis of QSAR‐based estimation of SSD parameters is that differences in ecotoxicity across chemicals are attributable to physicochemical differences across chemicals and that these differences can be quantitatively described by a (QSAR) model operating at the level of species assemblages. We investigated whether different combinations of physicochemical characteristics can be used to predict the SSD parameters (ecotoxicity) of nontested compounds by applying a QSAR‐type approach to the SSD parameters of data‐rich compounds.

We made a preliminary investigation to assess whether and to what extent the variability of the 2 parameters of log‐normal SSDs across chemicals—the median (μ) and the variance (σ^2^) of toxicity endpoints such as the no‐observed‐effect concentration (NOEC) and the median effect concentration (EC50)—can be empirically related to different combinations of the chemicals’ physicochemical characteristics. With an eye on practical needs, easily obtainable parameters were selected for the latter, to judge the working hypothesis for compounds for which only very basic molecular data are available and to develop models for screening‐level assessments of chemical pollution. Limitations on ecotoxicity data are common when there are ecotoxicity concerns regarding chemicals of emerging concern.

The aims of the present study were 1) to provide insight into whether and with what precision the 2 parameters of the log‐normal SSD model (the median of the toxicity endpoints [μ] and their variance [σ]) can be empirically described as a function of physicochemical characteristics, in the context of providing SSDs for, in principle, all compounds, and 2) to evaluate the potential utility of the resulting QSAR‐based SSDs. The exploration specifically considered freshwater ecotoxicity, given the array of curated ecotoxicity data for training the QSAR SSDs and in view of the European Union policy aims of reaching nontoxic surface water systems (Brack et al. 2015, 2017; European Union 2013; Posthuma et al. 2019). Our approach differs from other combinations of the concepts of QSARs and SSDs. For example, Van Leeuwen et al. (1991) proved that QSAR models may be useful tools in predicting toxicity endpoints for single species, to add species data to the test set required for deriving an SSD. Aldenberg and Rorije (2013) showed that combining single‐species toxicity endpoints based on QSAR estimates with toxicity measurements in a single SSD resulted in robust hazardous concentration for 5% of the species (HC5) estimates. Studies such as those of Belanger et al. (2015) show how results of such approaches are used in practice. Our working hypothesis represents another combination of QSAR and SSD concepts. The present study is the first to directly estimate the SSD parameters μ and σ for nontested compounds from known values from data‐rich compounds. Rather than predicting toxicity endpoints to expand on the species‐specific ecotoxicity data for a compound (the cited methods), our method aims at directly predicting SSDs for nontested compounds: QSAR‐based SSDs (derived from known SSDs).

## MATERIALS AND METHODS

### Overview of steps

The present study consisted of the following steps: 1) selecting a database encompassing SSD parameters for a large number of compounds, 2) selecting physicochemical characteristics and adding them to the database, 3) assessing empirical relationships between ecotoxicity and one or more of these characteristics (deriving QSAR‐based SSDs), and 4) evaluating robustness and model performance of the derived models by means of 4‐fold cross validation.

### Ecotoxicity database

As a starting point, we selected the database on SSDs for 12 386 compounds. The SSDs in that database are summarized in the formats of EC50‐based SSDs (based on acute EC50 data) and NOEC‐based SSDs (based on chronic NOEC data) by applying a log‐normal model to a large data set of ecotoxicity data (Posthuma et al. 2019). These are the original SSDs. Specifically, the starting material consisted of the log‐normal SSD parameters μ (median toxicity) for 12 214 and 7540 chemicals based on acute EC50s and chronic NOECs, respectively, and σ (variation in toxicity) for 9503 and 6864 chemicals, respectively. The NOEC SSDs in the present study were operationally based on EC5, EC10, EC20, or lowest‐observed‐effect concentration values, whereas ECs and lethal concentrations ranging from 30 to 70% were operationally marked as acute EC50s (Posthuma et al. 2019). The original SSD database also contains a quality score for each original SSD, to acknowledge differences in underlying data types and quantities. High‐quality original SSDs (based on measured data only, including a sufficient number of taxa) were used to train our QSAR‐based SSDs and moderate‐quality ones for testing our derived QSAR‐based SSDs (quality scores as in Table 1).

**Table 1:** Classification of species sensitivity distributions (SSDs) from Posthuma et al. (2019) into the high‐ and moderate‐quality subsets for training and testing quantitative structure–activity relationship‐based SSDs, respectively^a^

Quality criterion	High‐quality SSD data	Moderate‐quality SSD data
SSD fullness	Data on full SSD available (μ and σ)	Data on full SSD available (μ and σ)
Taxonomic coverage	Data on at least 10 taxa available	Data on 5–10 taxa available
Data origin	Measurements	Measurements and data extrapolated from other endpoints

^a^ The quality classes were assigned with 3 criteria: completeness of the SSD, taxonomic coverage, and the origin of the ecotoxicity data underlying the SSD (see Posthuma et al. 2019 for details).

### Physicochemical characteristics

The original SSD database was supplemented with selected physicochemical characteristics (Table 2), which served as predictors for the QSAR‐based SSD model derivation. In view of the practical applicability of QSAR‐based SSDs, ease of obtaining the characteristics was a key selection criterion. Where possible, values for these chemical characteristics were taken from Posthuma et al. (2019). If no value was available, a value (experimentally based) was taken from EPI Suite™, Ver. 4.18. Compounds lacking one or more of the predictors were omitted from model training and validation.

**Table 2:** Physicochemical characteristics used in the modeling

Description	Abbreviation	Unit	Detail	Sources for data imputation
Log_10_‐transformed octanol–water partition coefficient	*K*_OW_	—		KOWwin, Ver 1.68
Log_10_‐transformed water solubility	*S*	mg L^–1^		wskowWIN, Ver 1.42
Log_10_‐transformed vapor pressure (at 25 °C)	*VP*	mm Hg		MPBPWIN, Ver 1.42
Molecular weight	*MW*	—		wskowWIN, Ver 1.42.
Biodegradability	*biodeg*	—	^a^	BIOWIN, Ver 4.0
Functional groups	*func*	—	^b^	ECOSAR, Ver v1.11

^a^ Dummy variable representing the primary biodegradation classification of the chemicals, expressed in hours, hours–days, days, days–weeks, weeks, weeks–months, months, or recalcitrant.

^b^ Only the 6 most common functional groups were included: neutral organics, esters, phenols, aliphatic amines, acrylates, and inorganic compounds.

### Deriving QSAR‐based SSDs

The QSAR‐based SSDs were derived by fitting the following conceptual model to the high‐quality training data:

(1) $$\log\;y=f\left(K_{\mathrm{ow}},S,\mathrm{MW},\mathrm{VP},\mathrm{biodeg},\mathrm{func}\right)$$

This was done separately for the NOEC‐based SSDs and EC50_‐_based SSDs to separately estimate both μ and σ (represented by *y* in equation (1)). To enable determination of the relative contribution of each descriptor to the model, prior to the derivation of the QSAR SSD model, each continuous predictor was standardized, according to

(2) $$z_{i}=\frac{x_{i}-\overset{®}{x_{i}}}{s_{i}}$$

transforming the overall mean of physicochemical descriptor *i* ($\overset{®}{x_{i}}$) to 0 and the corresponding standard deviation (*s*_*i*_) to 1 (Eriksson et al. 2003).

We derived 2 multiple regression models (for μ and for σ, respectively) that incorporated the full set of 6 physicochemical parameters (Table 2), using the *lm* function in R, Ver. 3.5.1 (R Development Core Team 2013). To construct uncomplicated models for screening‐level impact assessments, no interactions or quadratic functions were included in model derivation. Then, the most parsimonious models for μ and σ were selected using the *dredge* function in R statistics, Ver. 3.5.1, based on the corrected Akaike information criterion (AIC) as well as the adjusted *R* ^2^. Afterward, the most influential predictors of ecotoxicity were identified by standardizing the regression coefficients using the *Relaimpo* package R statistics, Ver. 3.5.1.

### Robustness and validation of QSAR‐based SSDs

Multicollinearity was evaluated by calculating the variance inflation factor (VIF) of each input variable (as in Table 2), followed by an evaluation in which VIFs <5 were judged as absence of potential overfitting. Furthermore, the normality of the model residuals was checked by plotting them in a Q–Q plot. The QSAR‐based SSDs were internally validated by performing a 4‐fold cross‐validation test and externally validated by applying them to the moderate‐quality original SSDs from Table 1 (Veerasamy et al. 2011). All aforementioned tests were performed in R statistics, Ver. 3.5.1 (R Development Core Team 2013).

## RESULTS

### Original SSD parameters database

The initial database with SSD parameters of tested compounds used for training and testing represents a set of SSDs for 12 386 compounds of different qualities (Posthuma et al. 2019). Applying the criteria to select high‐quality SSDs resulted in subsets of EC50‐ and NOEC‐based SSDs for 616 and 92 compounds, respectively. Selection on moderate‐quality SSDs similarly resulted in 193 and 538 compounds, respectively. The complete subset (.xlsx) can be found in the Supplemental Data.

### Physicochemical characteristics

On adding the physicochemical characteristics, our database of SSDs for training and testing QSAR‐based SSDs was reduced to 299/86 (high quality/moderate quality) EC50‐based original SSDs and 37/246 SSD NOEC‐based original SSDs with complete data. These numbers of data were considered adequate for the exploratory purposes of the present study, given the relatively low number of predictors used (maximum 6).

### Empirical modeling

The coefficients of the formulae describing the empirical association between the values of the molecular descriptors of the compounds and the associated SSD parameters are shown in Table 3. Apart from the QSAR‐based SSD σ based on NOECs, all formulae were highly significant (*p* < 0.001). This implies that the null hypothesis—that the model coefficients equal zero (implying values of μ and σ not related to physicochemical characteristics of the compounds)—should be rejected. The idea to obtain ecotoxicity information for nontested compounds by QSAR‐based SSDs has merit, be it that the relationships are statistically significant (apart from the estimate of σ from SSD NOECs) but at the same time not yet very precise for non‐screening‐level assessment (further discussion in the next section). The highest adjusted *R* ^2^ values (and the lowest AICs) were found for models associated with NOECs rather than EC50s. However, models explaining the QSAR‐based SSD σ for both endpoints performed poorly (*R* ^2^ < 0.4). The relative importance analysis reveals solubility (log *S*) as the most important parameter influencing ecotoxicity (Supplemental Data, Figure S1), accounting for 36.6 and 38.8% of all variance in μ for EC50s and NOECs, respectively. Solubility in water appeared to be positively associated with the variation in μ for both EC50 and NOEC modeling, implying that the species’ sensitivity toward a chemical decreases with increasing solubility. Of approximately equal importance (based on their *z* scores), the outcome shows a significant inverse relationship between primary biodegradability and the μ of EC50 modeling, implying that species sensitivity is higher for compounds that are less degradable (i.e., those with primary biodegradability classified as weeks or longer). In the present study, adding molecular weight in the linear model did not increase the predictive power, in contrast to results from earlier work (Struck et al. 2008).

**Table 3:** Empirical relationships between species sensitivity distribution model parameters (μ and σ) and selected predictors for the z‐transformed parameters and untransformed parameters^a^

SSD parameter	*R* ^2^	AICc	p	Intercept	log *S*	log *VP*	log *K*_OW_	Hrs	Hours–days	Days	Days–weeks	Weeks	Weeks–months	Months	Recalcitrant	Aliphatic amines	Esters	Inorganic compounds	Neutral org.	Phenols
*z‐transformed parameters*																				
Acute EC50																				
Mu	0.56	629.2	<0.0001	**3.91**	**0.77**	**0.19**	–0.02		0.25		–0.21	**–0.61**	**–0.87**	**–0.35**	**–0.61**		–0.79	–0.11	0.3	–0.322
Chronic NOEC																				
Mu	0.72	82.6	<0.0001	**1.87**	**0.82**	–0.062			–0.2		–0.34	–0.22	–0.74	0.55			–0.08	1.67	0.66	–0.01
*Untransformed parameters*																				
Acute EC50																				
Mu	0.56	878.9	<0.0001	**3.4**	**0.29**	0.033	–0.007		0.25		–0.21	**–0.61**	**–0.87**	**–0.35**	**–0.61**		–0.79	–0.11	0.3	–0.322
Sigma	0.29	161.9	<0.0001	**1.008**	**–0.063**	–0.005	**–0.036**		–0.14		**–0.101**	–0.012	0.066	–0.19	0.02		0.272	0.018	0.014	–0.084
Chronic NOEC																				
Mu	0.72	108.7	<0.0001	**1.21**	**0.3**	–0.01			–0.2		–0.34	–0.22	–0.74	0.55			–0.08	1.67	0.66	–0.01
Sigma	0.38	52.31	0.422	0.34	0.045	0.028	–0.012		–0.21		–0.27	–0.38	–0.34	–1.02			0.241	0.429	0.377	0.064

^a^ To enable ranking the relative importance of the descriptors and for application in practice, respectively.

Significance of the individual parameters is indicated in bold.

AIC = Akaike information criterion; EC50 = median effect concentration; NOEC **=** no‐observed‐effect concentration; SSD = species sensitivity distribution.

**Figure 1:**
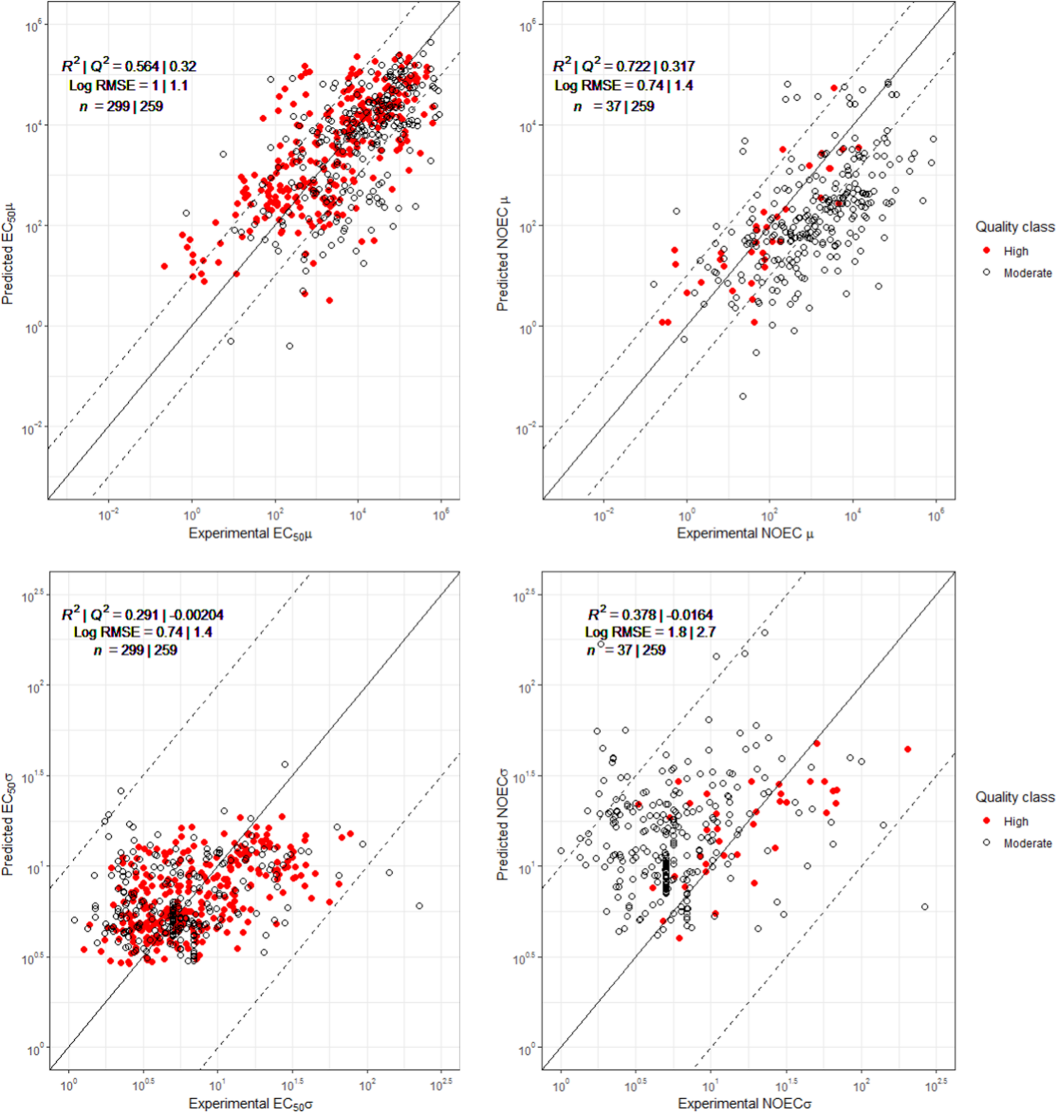
Illustrating the robustness (considering the significance [slope] and the precision [variability of *Y* for a given *X*] of the models) of the model‐predicted μ and σ (upper and lower figures, respectively) given the species sensitivity distribution parameters of tested compounds. The predicted toxicity endpoints (*Y*) were plotted against the measured ecotoxicity parameters (*X*) for both the high‐quality data set (in red) and the moderate‐quality data set (in black). Corresponding root mean square errors, *R* ^2^ values, and sample size for the training set and test set are shown. The line represents the 1:1 relationship of *X* and *Y*, and dashed lines represent a factor of 10 under‐ or overestimation by the model. RMSE = root mean square errors; EC50 = median effect concentration; NOEC = no‐observed‐effect concentration.

A comparison of the QSAR‐based SSD fits for μ between the NOEC‐ and the EC50‐based models showed that the former performed better than the latter (higher adjusted *R* ^2^ values; Figure 1). Despite lower input data numbers for chronic NOEC SSDs, the QSAR SSD for this endpoint fits relatively well (*R* ^2^ = 0.72) to the available data.

### Robustness and validation of QSAR‐based SSDs

The VIFs calculated for the predictors used in the QSAR‐based μ and σ models (for both EC50‐ and NOEC‐based models) were mostly below 5, except for the nonsignificant SSD NOEC σ estimation (Supplemental Data, Table S1). For the significant models, this indicates limited multicollinearity between input variables and a limited potential for interpretation error. Residuals of the QSAR‐based estimates of the QSAR‐based μ values were normally distributed, albeit heavy‐tailed, for EC50‐ as well as NOEC‐trained models (Supplemental Data, Figure S1). Averaged RMSEs calculated in the 4‐fold cross‐validation analysis (Supplemental Data, Figure S2) were lowest in the models for μ and σ associated with acute EC50 values as training set (1.23 and 0.131, respectively). Differences between model *R* ^2^ values, pertaining to the training data set, and calculated Q^2^ value, pertaining to the model fit on the test data set, were much higher for models pertaining to NOECs than for models pertaining to EC50s (Figure 1). However, because differences between *R* ^2^ values and *Q* ^2^ values were relatively high for models for both EC50s and NOECs, this may indicate possible overfitting.

## DISCUSSION

The present study resulted in the derivation of models to predict the 2 parameters of log‐normal SSDs (μ and σ) from ecotoxicity data of data‐rich compounds combined with easily obtainable molecular characteristics. Their potential use asks for an evaluation, and possible improvement, of the significance of the relationships as well as the precision of the predicted toxicity when applied in practice for nontested compounds. The models showed high statistical significance (*p* < 0.001, linear multiple regression) for SSD μ (median sensitivity of species), indicating that the slopes of the regression lines for the chosen set of physicochemical characteristics are greater than zero: the idea of QSAR‐based SSDs has merit to predict ecotoxicity of nontested compounds. Although the models explained 56 and 72% of the medians of SSDs based on EC50s and NOECs, respectively, variation in the medians of the SSDs explained in the external data set by our model (based on moderate‐quality data) was much lower for both SSDs based on EC50s and NOECs (31 and 32%, respectively), implying that these models perform worse when applied to compounds for which less ecotoxicity data were available. Because the out‐of‐sample predictive power for both SSD NOECs and SSD EC50s was much lower, this may suggest overfitting of the model in the case of modeling SSD parameters. Regarding prediction precision, although the present study showed that mainly the variance in the SSD parameter μ can be explained by a nontested compound's common physicochemical characteristics, more research on models with additional descriptors is necessary to derive accurate user‐oriented models to estimate SSD parameters. So far, the utility of the models would be screening‐level lower‐tier assessments which can discriminate low and high relative toxicity rather than precise prediction of molecularly relative closely related compounds.

In the present study no information is included on the exact endpoint tested per individual data record. Because all data entries of the data set used in construction of the model can refer to a variety of effects, it is uncertain to what extent the composition of tested endpoints is similar among the 2 training data sets for NOECs and EC50s. Because acute EC50s represent a set of similar adverse outcomes, whereas chronic NOECs may represent a wide variety of effects, we would expect NOECs to be less easy to predict than EC50s. The association for the shape parameter of the SSD (σ, across‐species sensitivity variation) was much lower. This was to be expected because interspecies differences in ecotoxicity are thought to originate from differences in physiology and metabolism between species, rather than differences in molecular properties of chemicals. Because QSAR‐based SSD σs are highly related to interspecies and interindividual correlations, diversity in species and individuals plays a large role in the observed variation and thus the prediction of σ. Because some compounds have a very species‐specific toxic mode of action, the accuracy of predicted σ values strongly depends on the taxa represented in the training set. Although in the present study this was mitigated by truncating data based on taxonomical coverage (Table 1), the exact distribution of species types within the model was unknown. In addition, QSAR‐estimated σ values likely follow a gamma instead of a normal distribution, highly skewed to the right. Some studies state that for each Gaussian predictor variable, the dependent variable should be normally distributed. In the present study, all continuous predictor variables were (log‐)normally distributed, implying the need for a Gaussian dependent variable. If the inherent problem of estimating σ would be solved, further efforts to model σ values would require a different way of modeling (Furman 2008). In the present study we described how ecotoxicity for species assemblages can be approximated using physicochemical descriptors for nontested compounds, resulting in models that were, moreover, significant and relatively most robust for SSD μ. However, we consider that this is only a first approximation of the models that can be derived and that can serve as QSAR‐based SSDs in environmental protection, assessment, and management. For SSD models lacking an estimate for σ, an intermediate slope may be adopted, based on the average slope of all ecotoxicity data (0.71), as reported by Posthuma et al. (2019). Overall, in the majority of substances for both the SSDs based on acute EC50s and chronic NOECs (>65%), adoption of this intermediate slope resulted in computed SSDs that were statistically similar (*p* > 0.05, Student *t* test) to the SSDs of the training set (Supplemental Data, Figure S3). However, the slopes may differ among toxic modes of action and chemical classes and may need further investigation to obtain appropriate average slopes per chemical class or mode of action, suitable for environmental risk assessment.

The set of molecular characteristics used so far was selected to merge utility criteria on the final SSD models with availability criteria on the characteristics. For the selected set, solubility was the strongest predictor of toxicity, with decreasing solubility leading to higher toxicity. This finding was supported by Mayer and Reichenberg (2006) and Escher et al. (2017), indicating that aquatic baseline toxicity, up to a certain cutoff point, increases with decreasing solubility. Albeit in the present study no significant relationship was found between log *K*_OW_ and log‐transformed toxicity endpoints, multiple studies suggest that the log *K*_OW_ may be a stronger predictor for toxicity than solubility because this physicochemical characteristic may act as a proxy for *K*_lipid membrane–water_ (Endo et al. 2011; Escher et al. 2017), up to a cutoff point of approximately 6 (Könemann 1981; Escher et al. 2017).

Elaboration of the QSAR approach to estimate SSD parameters from known SSDs to improve both significance and potential ecotoxicity prediction precision can be further substantiated by considering science‐based criteria to derive the QSAR‐based models. A logical expansion of the working hypothesis would, for example, consider updating the model using mechanism‐related chemical descriptors (e.g., topological descriptors) and could, in addition, include certain traits of tested species in model derivation. The latter would yield QSAR‐based SSDs for separate taxonomic groups with an expectedly improved precision, given the fact that molecular characteristics embodying, for example, “insecticidal action” relate to “insect species traits.”

To optimize their utility, QSAR‐based SSDs should be statistically significant and precise (i.e., based on sufficient, reliable, and meaningful data and data types). A mere addition of trait‐specific predictors has, however, various consequences on the statistical power or on limitations such as on obtaining descriptor data for all chemicals in the study data set (loss of data in the “training set” for modeling). Conflicting (statistical) effects may occur, and conceptual improvements may trade off with statistical power and utility of results. In the present study, this bias was overcome by using the AIC scores. Further derivation of these models asks for a balance between the science‐based expansion of (molecular and/or species trait) descriptors and the opportunity to (easily) obtain descriptor data, to potentially use the net outcomes of this approach in the format of QSAR‐based SSDs in decision‐support processes for environmental protection, assessment, and management.

The models derived in the present study could potentially serve as a screening‐level exploration into the potential of chemicals to pose harm to ecosystems via direct effects on species assemblages for those chemicals lacking in ecotoxicity data. For such chemicals, the QSAR‐based SSDs would enable translation of molecular characteristics into the μ and/or σ of associated SSD models. We note, however, that to be helpful to derive exploratory protective benchmarks (e.g., HC5_NOEC_) or exploratory toxic pressures (potentially affected fraction) of observed environmental concentrations, there is a need for an improvement of the QSAR‐based approach to derive improved slope parameters (σ). Protection from chemical pollution, and the related assessment and management of these chemicals, hinges on approaches for their prioritization. Chemicals can be prioritized using protective benchmark concentrations based on chronic NOECs or similar endpoints. Protective benchmarks have been derived for some thousands of chemicals worldwide because of the importance of benchmark concentrations for achieving a level playing field for a jurisdiction such as the European Union to judge for allowing chemicals on the market or to initially judge environmental quality. Only a fraction of the 100 000+ compounds in commerce are currently evaluated regarding potential impacts so that the potential impact of mixtures of these chemicals cannot be evaluated, often resulting in neglect. Both QSAR modeling as well as SSD modeling are generally used toxicity extrapolation methods in environmental risk assessment. However, predicting ecotoxicity based on physicochemical characteristics or structural alerts is mainly done for only one species or one substance at a time, requiring extensive data on toxicity as well as on the predictive variables in the model, to provide a statistically sound model (Dearden 2002). The approach of deriving and using QSAR‐based SSDs provides a fully novel concept to fill this gap, in line with pleas such as those of Hendriks (2013) and Brack et al. (2017), who replied to associated global societal needs and concerns on water pollution (Bernhardt et al. 2017). Although uncertainties regarding the first resulting QSAR SSDs may be high (Table 3), the variation among the SSD‐μ value of all tested substances is much higher (see Supplemental Data, Figure S3), making the QSAR‐based SSD approach suitable in light of first‐tier chemical prioritization, especially “inverse prioritization,” identification of compounds with a low probability of ecotoxicological impact (compounds that locally do not exert effects on species assemblages via direct impacts on growth and reproduction; Posthuma et al. 2019). The chance of the 10% least toxic compounds exceeding the 10% most toxic compounds is negligible (*p* < 0.05; Supplemental Data, Figure S3).

We consider it highly relevant to assist focusing policy and management attention for a problem consisting of 100 000+ chemicals and their mixtures by providing a method—QSAR‐based SSD modeling—that can help in identifying the compounds for which it is highly unlikely that they pose harm by direct effects of species in species assemblages. Given our earlier findings (Posthuma et al. 2019), we conclude that we found the derivation of QSAR SSDs feasible and their use in environmental protection, assessment, and management potentially profitable.

## Supplemental Data

The Supplemental Data are available on the Wiley Online Library at DOI: https://doi.org/10.1002/etc.4601.

## Supporting information

This article includes online‐only Supplemental Data.

Supplementary Material

Supplementary Material

## Data Availability

Data, associated metadata, and calculation tools are available from the corresponding author (r.hoondert@science.ru.nl). Data pertaining to this article are located at figshare (https://wiley.figshare.com/etc).
