# Supplementary material for: QSAR‐Based Estimation of Species Sensitivity Distribution Parameters: An Exploratory Investigation
Source: Environ Toxicol Chem. 2019 Nov 9;38(12):2764–70. doi: 10.1002/etc.4601 (PMC6900027; doi:10.1002/etc.4601)
Supplement: Supplementary file 1 — Supplementary Material [file ETC-38-2764-s001.docx]

**SUPPORTING INFORMATION:**

**QSAR-based estimation of SSD parameters – an exploratory investigation**

**SHORT COMMUNICATION**

Renske Hoondert ^1,2^

Rik Oldenkamp ^2^

Dick de Zwart ^3^

Dik van de Meent ^2, 3^

Leo Posthuma ^1,2 *^

^1^ RIVM, Centre for Sustainability, Environment and Health, P.O. Box 1, 3720 BA Bilthoven, the Netherlands.

^2^ Radboud University Nijmegen, Department of Environmental Sciences, Faculty of Science, Nijmegen, the Netherlands

^3^ ARES, Odijk, the Netherlands

**Running head:**

QSAR-based estimation of SSD parameters

^*^ Corresponding author. To whom correspondence may be addressed.

Table S1: Calculated variance inflation factors (VIFs) quantifying the severity of multicollinearity in an ordinary least squares regression analysis for the four models derived in the present study, in which VIFs lower than 5 were judged as absence of potential interpretation bias.

|  | SSD-EC50-μ | SSD-NOEC-μ | SSD-EC50-σ | SSD-NOEC-σ |
| --- | --- | --- | --- | --- |
| sol | 4.63 | 2.66 | 4.63 | 6.14 |
| Kow | 4.73 |  | 4.73 | 7.8 |
| VP | 1.34 | 2.06 | 1.34 | 7.77 |
| biodegdays_weeks | 1.7 | 1.97 | 1.7 | 2.26 |
| biodeghours_days | 1.13 | 1.17 | 1.13 | 3.58 |
| biodegmonths | 1.08 | 1.32 | 1.08 | 1.27 |
| biodegrecalcitrant | 1.08 |  | 1.08 | 2.93 |
| biodegweeks | 1.83 | 1.51 | 1.83 | 2.44 |
| biodegweeks_months | 1.27 | 1.72 | 1.27 | 2.36 |
| funcEsters | 4.76 | 4.31 | 4.76 | 6.82 |
| funcInorganic_Compound | 2.15 | 2.14 | 2.15 | 2.91 |
| funcNeutral_Organics | 4.67 | 4.05 | 4.67 | 6.37 |
| funcPhenols | 2.34 | 2.81 | 2.34 | 4.76 |


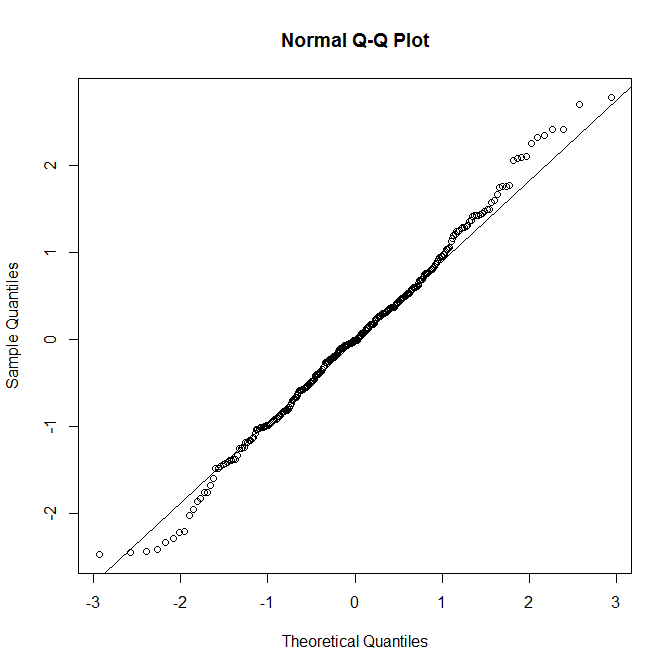

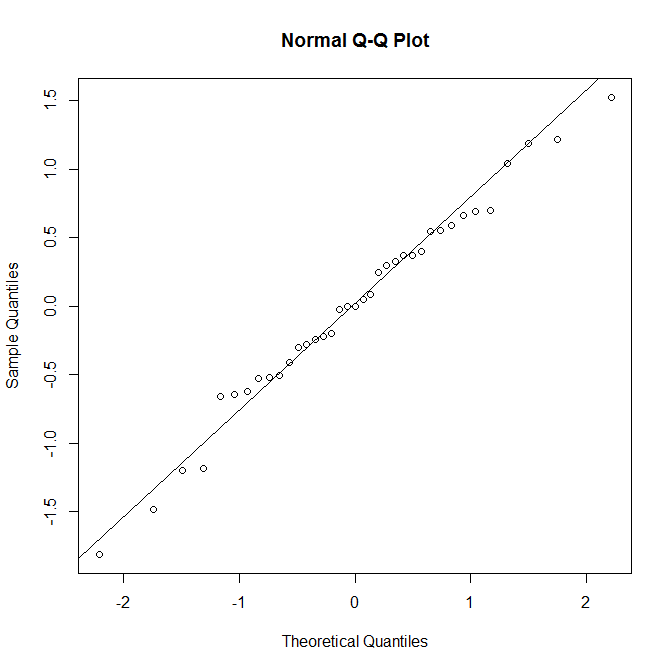

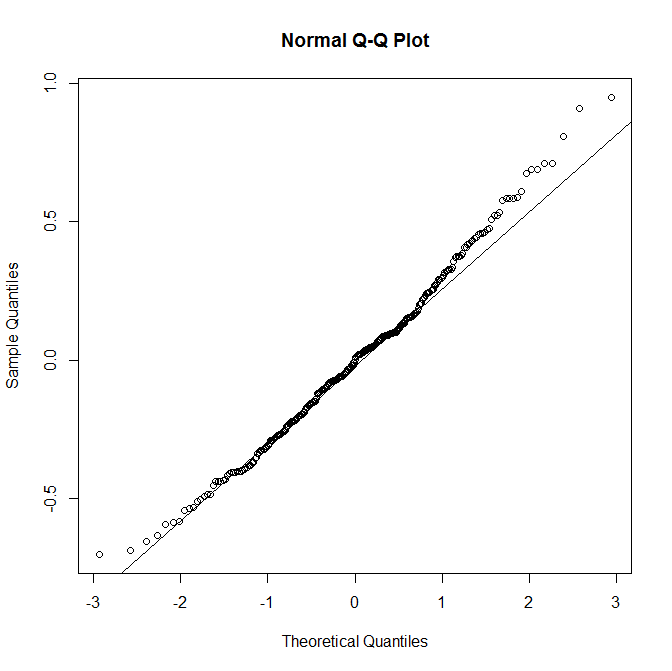

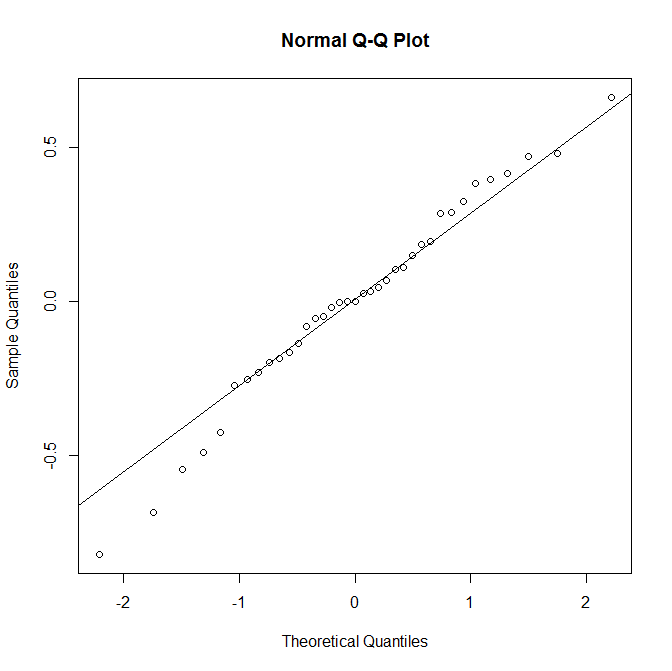


**Figure S1: Normal Probability Plot of the Residuals for models pertaining to QSAR-SSD-μ (upper plots) and QSAR-SSD-σ (lower plots) for acute EC_50_s (left plots) and chronic NOECs (right plots), repectively.**


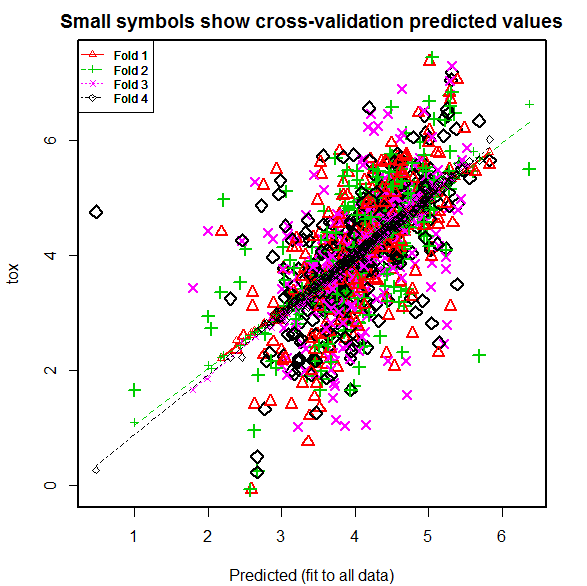

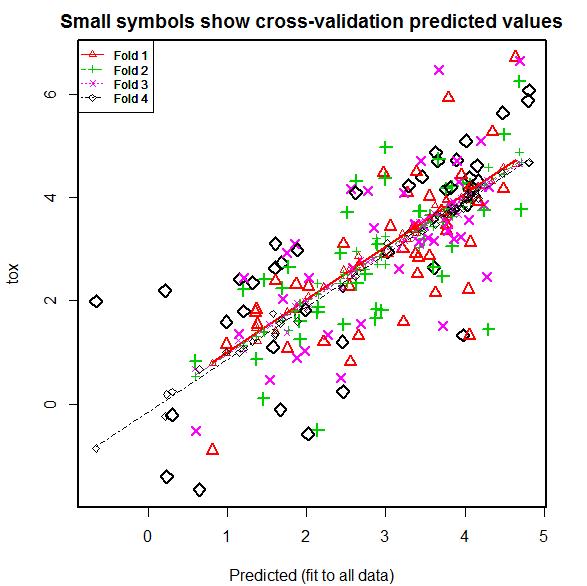

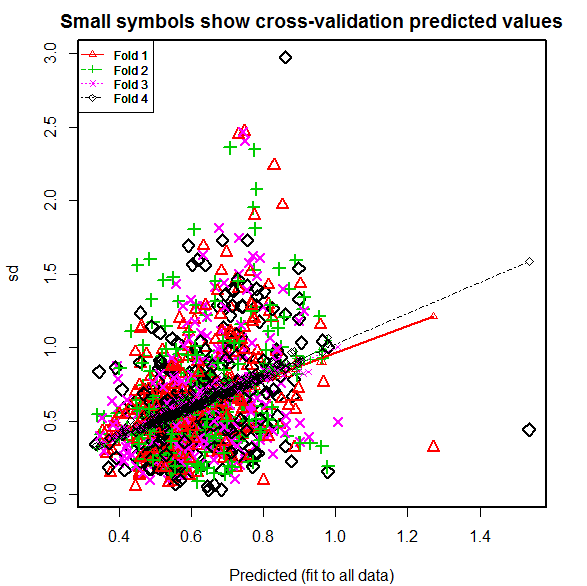

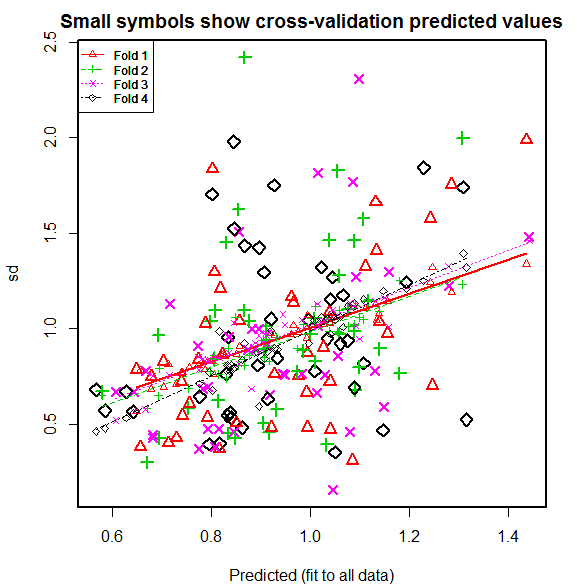


Figure S2: 4-fold cross validation result plots for models derived in this study (clockwise, starting at the upper left): QSAR-SSD-μ for acute EC_50_s, QSAR-SSD-μ for chronic NOECs, QSAR-SSD-σ for chronic NOECs and QSAR-SSD-σ for acute EC_50_s.


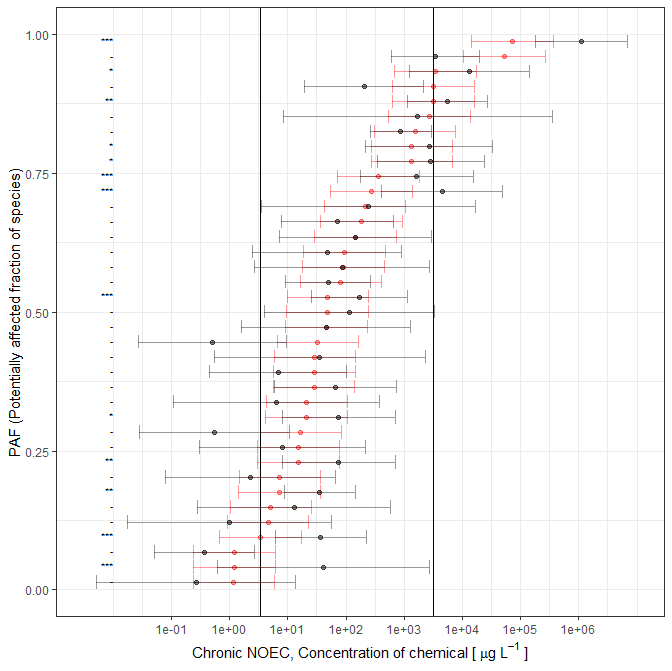

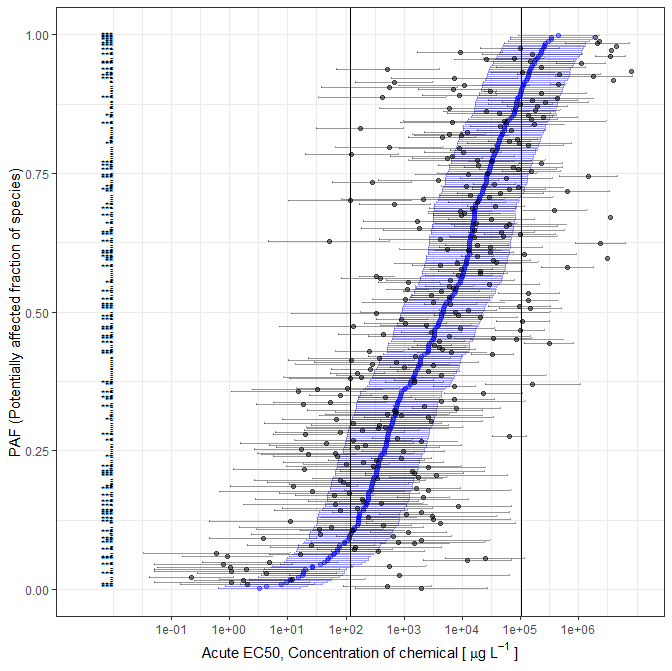


Figure S3: Cumulative distribution of μ’s (in blue/red) and corresponding estimates (in black, for both high quality data) for acute EC_50_s (left) and chronic NOECs (right). The vertical lines indicate the 10% most toxic and least toxic substances respectively. The number of stars indicate the significance level between the measured μ and σ and estimated μ (σ = 0.7): ***: P < 0.001, **: P < 0.01, *: P < 0.05, -: N.S.).
